# The Challenge of Home Allergen Re-introductions Using the Ladder Approach in Children With Non-IgE Mediated Gastrointestinal Food Allergy

**DOI:** 10.3389/falgy.2021.721686

**Published:** 2021-09-10

**Authors:** Rosan Meyer, Claire De Koker, Robert Dziubak, Heather Godwin, Kate Reeve, Adriana Chebar-Lozinsky, Ru-Xin Foong, Ana-Kristina Skrapac, Marlene Ellmer, Neil Shah

**Affiliations:** ^1^Department of Paediatric, Imperial College, London, United Kingdom; ^2^Brackengate Intermediate Care Facility, Cape Town, South Africa; ^3^Department of Gastroenterology, Great Ormond Street Hospital NHS Trust, London, United Kingdom; ^4^Frimley Health NHS Foundation Trust, Camberley, United Kingdom; ^5^Royal Hospital for Sick Children and Young People, Edinburgh, United Kingdom; ^6^Allergy Immunology, Murdoch Children's Research Institute, Melbourne, VIC, Australia; ^7^Department of Women and Children's Health (Paediatric Allergy), School of Life Course Sciences, Faculty of Life Sciences and Medicine, King's College London, London, United Kingdom; ^8^Children's Allergy Service, Evelina London Children's Hospital, Guy's and St. Thomas' NHS Foundation Trust, London, United Kingdom; ^9^Department Nutrition and Dietetics, Chelsea and Westminster Hospital NHS Foundation Trust, Department of Nutrition and Dietetics, London, United Kingdom; ^10^Department of Gastroenterology, Great Ormond Street Hospital NHS Trust, London, United Kingdom; ^11^Department of Gastroenterology, University College London, UK and Reckitt, Slough, United Kingdom

**Keywords:** non-IgE mediated food allergy, home introduction protocol, milk ladder, tolerance to food allergens, safety of home introductions

## Abstract

**Background:** Oral food challenges remain the most reliable method for allergy confirmation. Although consensus guidelines have been published to unify Immunoglobulin E (IgE)-mediated challenges, this does not exist for non-IgE mediated gastrointestinal allergies outside of Food Protein Induced Enterocolitis Syndrome. We therefore set out to establish the use of home introduction protocols (HIP) for confirmation of food allergy for milk, soya, egg and wheat using a ladder approach in children with non-IgE mediated allergy.

**Materials and Methods:** Patients with suspected non-IgE mediated gastrointestinal allergies (0–16 years) were recruited following symptom improvement on an elimination diet. All children had skin prick or specific IgE tests to rule out IgE-mediated allergies prior to suggestion the HIP. Number of trials and outcome was documented. HIPs were developed using a published ladder approach for cow's milk as baseline and final dose was calculated based on guidelines for food protein induced enterocolitis syndrome and portions for age from the National Diet and Nutrition Survey. First foods were baked/highly processed and every 4th day patients moved to a more unprocessed/unheated food.

**Results:** From 131 recruited patients, 117 (89.3%) followed the HIP for food allergens. No adverse events were documented. In more than 50% of cases one attempt at the HIP was sufficient to establish allergy status, but many required 2–5 attempts before the outcome was clear. About half of the children were fully tolerant to foods they initially eliminated: 36, 26 and 30% were partially tolerant to milk, soya, and egg and only 15% achieved partial tolerance to wheat. Wheat was the allergen introduced earliest, followed by soya, cow's milk and egg.

**Conclusions:** This study indicates that home HIPs are safe in non-IgE mediated gastrointestinal food allergy and that the ladder approach may be useful in re-introducing allergens in children at home with non-IgE mediated gastrointestinal allergies. From this study we can also conclude that tolerance to processed/baked allergens was observed in many children. Further studies should be performed on the HIP and ideally reintroduction should occur pre-defined time intervals.

## Introduction

Food allergies in children can be Immunoglobulin-E (IgE), non-IgE mediated or mixed IgE/non-IgE mediated (1). IgE-mediated reactions usually occur within 2 h of ingesting the offending allergen and are well-defined both clinically and scientifically. Both skin prick tests and specific IgE blood markers are available to guide diagnosis, but oral food challenges (OFCs) remain the most reliable method for food allergy confirmation (2). In 2012, Sampson et al. (3) published consensus guidelines to unify practice on the execution and dosages of IgE-mediated OFCs, which are used as guidance by most allergy centers.

Non-IgE mediated allergies usually present at least 2–48 h after the ingestion of the offending food allergen but can take in some cases even longer for symptoms to be apparent and usually affects the gastrointestinal tract and/or skin (4). The delayed presentation of food allergy symptoms is very common, with data from the United Kingdom (UK) indicating that almost 60% of children with cow's milk protein allergy present with gastrointestinal symptoms and/or atopic dermatitis and the challenge proven incidence of non-IgE mediated cow's milk allergy in the UK was 0.73% (5, 6). The pathophysiology and diagnosis of food associated atopic dermatitis and IgE mediated allergy is much better understood, whereas non-IgE mediated food allergies affecting the gastrointestinal tract are clinically well-described but outside of eosinophilic oesophagitis and food protein induced enterocolitis syndrome the pathophysiology is not that well-established (7, 8). As such there is an absence of non-invasive tests supporting the diagnosis (7). Therefore, gold standard method for the diagnosis and confirmation of this delayed allergy remains an OFC (4). However OFCs are not well-established in non-IgE mediated food allergies and challenge standards have not been published due to the diverse time of onset, variety food amount required for a reaction to occur and wide spectrum of clinical symptoms (9). To date the only published standard OFC protocol for non-IgE mediated allergies is for Food Protein Induced Enterocolitis Syndrome (10). FPIES has a rapid onset, usually within 1–4 h after the ingestion of the food allergen, symptoms can be quite severe (i.e., profuse vomiting) and therefore an OFC for this diagnosis should occur in hospital. The symptoms for other non-IgE mediated gastrointestinal allergies usually have a longer time to onset, which makes it difficult and costly to admit children not only for hours but for days to hospital for an OFC, especially as there is an extremely low risk for an acute life-threatening event. In addition to the complexity of performing these OFCs, parents are often reluctant to challenge due to recurrence of symptoms which may take a longer time to resolve (8).

In 2013 Venter et al. (11) published a milk ladder approach for home reintroduction in non-IgE mediated cow's milk allergy, which has subsequently been updated in 2017 (12). This approach is based on the extent of milk heating and fermentation, which has an impact on both the conformational epitopes and peptide length (13, 14). In our experience this approach was better accepted by parents, who perceive this method of reintroduction as less likely to cause a severe reaction at home (15). We therefore set out to develop and implement home reintroduction protocols (HIP) in a cohort of children with non-IgE mediated gastrointestinal food allergies using the food ladder approach for all common allergens.

## Materials and Methods

### Subjects

A prospective, observational study was performed at Great Ormond Street Hospital NHS Foundation Trust, Gastroenterology Department, in the UK between November 2011 and August 2014. Ethical approval was obtained for this study (11/LO/1177). We invited eligible parents of children [without non-allergic co-morbidities (i.e., cerebral palsy, cardiac disorders)] aged 4 weeks to 16 years, with symptoms of a non-IgE mediated food allergy to take part in the study. Participants were required to follow an elimination diet for the diagnosis of suspected non-IgE mediated gastrointestinal allergies. All children received dietetic advice at the time of the elimination diet using the standard Food Allergy Specialist Group diet sheets from the British Dietetic Association. A 3-day food diary was completed by subjects if dietary elimination led to symptom improvement (after 4 weeks of elimination). This allowed for the assessment of nutritional intake but also compliance with dietary elimination. Dietary intake data has been published in two previous studies (16, 17). A Likert Scale Gastrointestinal Symptom Questionnaire was used to aid the initial diagnosis of non-IgE mediated gastrointestinal allergies. This questionnaire has been used in other studies that have been published by the same research group (18). The questionnaire was administered prior to starting the elimination diet and again at 4 and 8 weeks after starting the food elimination diet. If there was no improvement in symptom score at 4 weeks, the family continued with dietary elimination and repeated the questionnaire after a further 4 weeks (8 weeks total after commencing the elimination diet). Children were only enrolled in the study if symptoms scores improved with the dietary elimination at either the 4 or 8-week assessment. Children for this study were classified as non-IgE mediated allergy based on the success of the elimination diet and home reintroduction using the HIP and not on endoscopic evidence, as the majority did not undergo an endoscopic procedure, which is not routine practice in the UK.

### Home Introduction Protocols

HIPs were developed using the published milk ladder by Venter et al. (11) as baseline. We shortened the number of stages to ensure that the whole HIP for milk could be completed in about 2 weeks and used the same principles for developing HIP for egg, soya, and wheat (Supplementary Material). As there were no published guidelines on top protein dose for the HIPs for most non-IgE mediated food allergic conditions, we based our protocols on a combination of the FPIES challenge dosage published by Nowak-Wegrzyn et al. (10) and the National Diet and Nutrition Survey for the UK (https://www.gov.uk/government/collections/national-diet-and-nutrition-survey). This survey is based on a rolling programme that covers food consumption, nutritional intake and nutritional status from children and adults aged from 18 months upwards (*n* = 6,828 1–4 year-olds). It is complemented by a one-off diet and nutrition survey in 2011, which includes this data also for 4–18 month-old infants (*n* = 4,451). Dietary intake, including food portions were based on personal interview, a 4-day dietary diary, blood samples, and estimates of breast milk intake, fluid intake, and body composition. This allowed the authors of this study to establish normal portions consumed by children of that age for these allergens. Based on the 50th centile on the growth charts, we calculated 0.3–0.6 g/kg (10g maximum) of protein of the food allergen and compared this to the National Diet and Nutrition Survey to ensure that end dosages met what children would usually consume per day for that food allergen (16). The end doses therefore for all the age groups were ≥10 g of protein, which is the maximum dose suggested for a FPIES challenge. There were no specific portion recommendations for soya from the National Diet and Nutrition Survey, but we used the portions recommended for milk/milk products, as soya would commonly be used as cow's milk replacement. From the final dose, we worked backwards to provide 3-day staged food protein dosages (increased amount every 4th day) that started with foods being baked or processed and moved up the ladder to achieve the final unprocessed/unbaked dose. Parents were advised to provide foods throughout the day (not all at once) and food introductions were cumulative, so if one step was tolerated children continued to consume these foods as per normal, whilst adding in the next step (Supplementary Material). Where appropriate volumes were rounded up, to make the HIP more practical and easier for parents.

### HIP Procedure and Monitoring or Reactions

All children in our research cohort received a skin prick test (SPT) to milk, soya, egg, wheat, fish, and peanut (tree nuts SPT only if space on arm available) as a part of their research appointment and if this was not possible, or histamine control was negative, specific IgE's to foods were performed. No home introductions were performed in children that had a SPT > 3 mm or positive specific IgE to foods; these children were referred to an allergy center for further advice and supervised OFC. In addition, all children with known FPIES were also excluded from home reintroductions. Home introductions were commenced on the research gastroenterologist recommendation after a research clinic review (after at least 4–8 weeks elimination), if children were found to have improved symptoms and stable on the elimination diet. Parents received either face-to-face advice on how to perform home introductions or they received the same information via phone. This was accompanied with a written HIP that also stipulated that the HIP should only be commenced when the child was well. Additionally, all parents had antihistamines at home in case of more serious reactions, which the research team documented if they occurred. Home introductions were monitored by experienced pediatric gastroenterology research dietitians via phone and e-mail, using the previously published Likert Scale Gastrointestinal Symptom Questionnaire by our group (Supplementary Files) (18). If parents reported significant deteriorations in their child's gastrointestinal symptoms or worsening of eczema/respiratory symptoms, the involved gastroenterologist was consulted to decide whether the home introduction failed or whether a retrial was warranted. If any reported reactions were ambiguous, they were requested to re-start the HIP when the child was well again. The number of attempts were documented, but only the final successful home reintroduction results are reported in this publication. A child was classified as fully tolerant if they tolerated allergens in normal amounts usually expected in a child of that age, partially tolerant if they tolerated baked, fermented or a highly-processed form of the allergen and still allergic if none of the stages of the HIP were tolerated.

### Statistical Analysis

Data were analyzed with IBM SPSS Statistics for Windows, Version 22 (Armonk, NY). Continuous variables are presented as medians with interquartile ranges, and categorical variables are presented as proportions and percentages.

## Results

We identified 252 outpatients with suspected non-IgE mediated gastrointestinal food allergies between December 2011 and November 2013 (study continued until August 2015 to finish the HIP) that were eligible for inclusion in the study. Ninety-one patients were excluded because they did not want to partake in the study, were unable to attend, lost to follow up or had non-atopic co-morbidities. Therefore, 161 children were enrolled in the study, of which 30 patients did not improve on the elimination diet. They were excluded and subsequently diagnosed with functional gastrointestinal diseases or other gastrointestinal disorders (inflammatory bowel disease/coeliac disease). We therefore included data for 131 patients with a mean age of 22 months (IQR 7-66) at the time of enrolment, with their symptom presentation and improvement already published by Chebar Lozinsky et al. (18). The majority of children experienced symptom improvement on milk (24%) or milk and soya elimination diet (22.9%). A further 53.1% of children required additional elimination of egg, wheat and other allergens to improve gastrointestinal symptoms (14).

Of 131 patients, 117 (89.3%) followed the HIP for one or more of the allergens during the study period, of which 82/117 (70.1%) were male. Fourteen children (from original 131 patients) were excluded for the HIP due to specific IgE-sensitization, which included: 6/114 (5%) to cow's milk, 5/71 (7%) to egg, 1/70 (1%) wheat and 2/77 (2%) to soya. In addition, there were patients that were deemed too unstable to do the HIP for certain foods by the responsible gastroenterologist, and there were parents that refused the HIP, therefore not all 117 patients had all allergens introduced at home during the study period. Home introductions occurred in 92/114 (81%) children who eliminated cow's milk, 61/71 (86%) egg, 60/70 (77%) wheat and 75/100 (77%).

**Figure 1:**
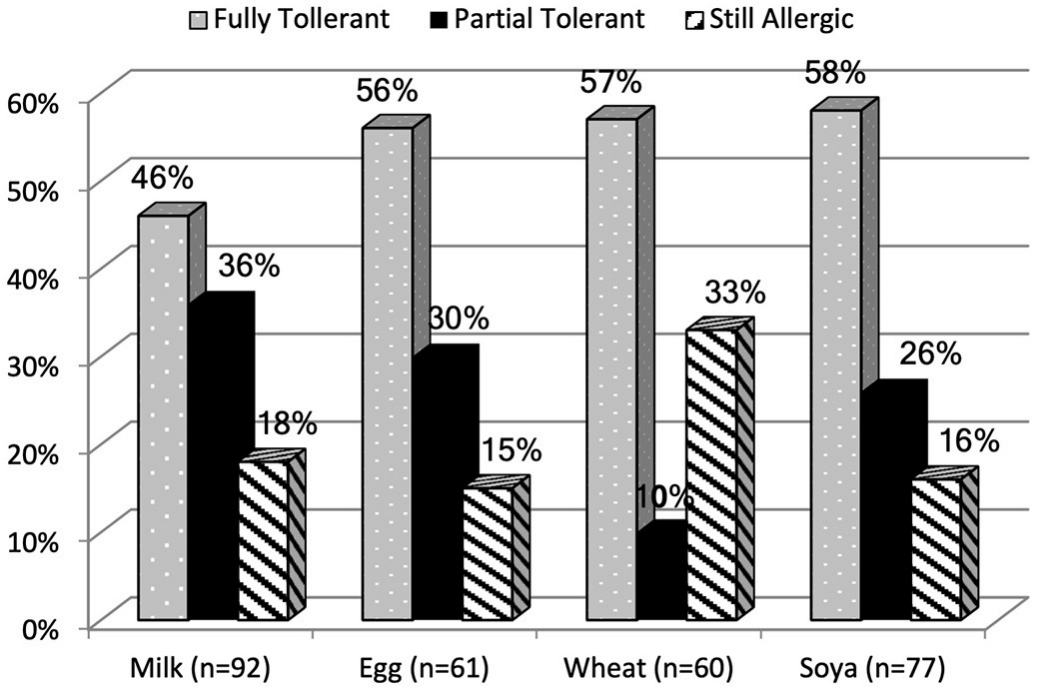
Outcome of HIP of allergens categorized as fully tolerant, partially tolerant and still allergic.

No serious adverse events were reported with the use of our HIPs. Our data indicated that between 46 and 58% of the children were fully tolerant to the food they were eliminating at the time of performing the HIP (Figure 1). In more than 50% of cases one attempt at the home introduction was sufficient to establish tolerance/reactivity to the challenge food, but 2–5 attempts were needed for the rest of the home allergen introductions to establish allergy status (Table 1). This was related to illness/teething occurring during the home introduction that led to ambiguous symptoms.

**Table 1:** Summary of HIP attempts and attainment of outcome.

	**Tolerance level**	**Number of attempts**	***N***	**% passed HIP**
Cow's milk	Fully tolerant (*N* = 42)	1	22	53
		2–5	17	40
		>5	3	7
	Partially tolerant (*N* = 33)	1	13	39
		2–5	13	39
		>5	7	22
	Still allergic (*N* = 17)	1	5	29
		2–5	8	47
		>5	4	24
Egg	Fully tolerant (*N* = 34)	1	19	56
		2–5	15	44
		>5	0	0
	Partially tolerant (*N* = 18)	1	6	33
		2–5	10	56
		>5	2	11
	Still allergic (*N* = 9)	1	0	0
		2–5	6	67
		>5	3	33
Wheat	Fully Tolerant (N = 34)	1	22	65
		2-5	11	32
		>5	1	3
	Partially tolerant (*N* = 6)	1	2	33
		2–5	4	67
		>5	0	0
	Still allergic (*N* = 20)	1	10	50
		2–5	7	35
		>5	3	15
Soya	Fully tolerant (*N* = 45)	1	35	78
		2–5	9	20
		>5	1	2
	Partially tolerant (*N* = 20)	1	10	50
		2–5	8	40
		>5	2	10
	Still allergic (*N* =12)	1	4	33
		2–5	6	50
		>5	2	17

We were also interested in the age of the children at the time of performing the home introduction as this occurred at different ages depending on age of inclusion in the study, severity of symptoms/symptom improvement and when the clinician deemed the child ready for the HIP. We calculated the median age and Inter Quartile Range (IQR) per food introduced and also what age children were fully or partially tolerant (Table 2).

**Table 2:** Mean age when children had the HIP and outcome categorized by tolerant, partially tolerant and still allergic.

	**Cow's milk—months [IQR^#^]**	**Soya—months [IQR]**	**Egg—months [IQR]**	**Wheat—months [IQR]**
Tolerant	17 [9–27]	13 [6–22]	18 [9–25]	9 [4–17]
Partially tolerant^*^	25 [16–35]	23 [18–2]	24 [17–28]	38 [30–47]
Still allergic	36 [24–47]	28 [16–22]	23 [21–27]	19 [11–28]

* *Defined as tolerant of baked/processed food containing the allergen*.

# *IQR, interquartile range*.

## Discussion

To the knowledge of the authors, this is the first publication aimed at establishing a HIP of food allergens, based on a food ladder approach in children with non-IgE mediated allergies outside of FPIES. A stepwise home approach for re-introduction of foods has been suggested for children with eosinophilic oesophagitis (EoE) guided by endoscopy, but to date the reintroduction procedures remain center specific based on experience and endoscopy protocols (19). The most important finding of our study was that HIPs were safe in this non-IgE mediated gastrointestinal cohort, and no serious adverse events were documented. However, all children that had IgE sensitization to foods or known FPIES were excluded. A shift from non-IgE mediated gastrointestinal allergies to IgE-mediated allergies has been described in FPIES and the overlap between IgE and non-IgE mediated reactions in EoE is also well-documented (20, 21). It is therefore our opinion, that it is important to take IgE-sensitization into consideration prior to suggesting home reintroduction for any child with a non-IgE mediated gastrointestinal food allergy, as 14 children from our cohort did show IgE-sensitization to common food allergens.

In about half of children only 1 attempt at the HIP was required. However, those with partial tolerance and who continued to display symptoms, multiple attempts at the HIP were required to confirm their allergy status. Data published by our group on the same cohort, indicated that although >98% of children showed overall symptom improvement, full symptom resolution did not occur in all patients; in particular, abdominal pain with back arching, flatus and food aversions did not fully improve. This means that many children may still have some symptoms whilst undergoing the HIP, which may complicate the assessment of tolerance/reactivity. In addition to lingering gastrointestinal symptoms, parents often reported frequent upper respiratory tract infections and teething as exacerbating factors in monitoring gastrointestinal symptoms during challenges. We have previously reported that almost 70% of our non-IgE mediated cohort had frequent upper respiratory tract infections with Latcham et al. (22) finding that 45% of their food allergic cohort had low IgA levels, with higher levels of low IgA in non-IgE mediated allergies, which may also reflect the younger age of the children. Development of such symptoms during home re-introduction will impact on the perception of symptom development. Teething has also been shown to lead to an increase in TNF-α, IL-8, IL-6, and IL1-ß, with TNF-α and IL1-ß linked to fever and sleep disturbances and IL1-ß and IL-8 to gastrointestinal disturbances (23). It is therefore important to repeat home introductions if results are ambiguous, in particular if confounding factors (as described above) occurred at the same time as food introductions. Postponing home reintroductions until a child experiences less frequent upper respiratory tract infections or has stopped teething in our opinion is not warranted as this would unduly postpone the reintroduction.

The mean age of children in this study was 22 months (IQR 7-66 months) at enrolment, reflective of a population referred for a second opinion at a tertiary referral center. Whilst the HIP protocol was initiated after 4–8 weeks elimination, many children were already above 1 year of age when they had the allergen reintroduced for the first time. This study found that 46% of children were fully tolerant at a median age of 17 months to cow's milk, 56% to egg at a median age of 18 months, 58% to soya and 57% were tolerant at 9 months to wheat. Vanto et al. (24) described tolerance in their non-IgE mediated cow's milk allergic cohort at 2, 3 and 4 years of age at 64, 92, and 98%. In our cohort a lower percentage of tolerance to cow's milk was reported at 17 months, but this may be related to the population being recruited from a tertiary referral center. Data on the development of tolerance in non-IgE mediated gastrointestinal egg allergy is not available and therefore difficult for us to compare our data to. From FPIES data on soya, Caubet et al. (25) found tolerance to soya in the majority of patients at a median age of 6.7 years, which is much older than our soya allergic cohort.

Around a third of children in our cohort at the time of home introduction were tolerant to some form of processed milk, egg, or soya, and to wheat (although partial tolerance to wheat was lower). Data from Nowak-Wegrzyn et al. (13) in 2008 indicated that 68% of children with a challenge confirmed IgE-mediated cow's milk allergy were tolerant to baked milk. In addition, a study by Alessandri et al. (14) showed 58% of children with an IgE-mediated cow's milk allergy tolerated a highly-fermented cheese (i.e., Parmigiano Reggiano®). Subsequent studies indicated that children who tolerated baked milk and had incorporated this in their diet, were 16 times more likely to become tolerant to unheated milk (26). Similar data in regard to outgrowing egg allergy has been published and has led to a paradigm shift in the management of IgE-mediated milk and egg allergy (27, 28). However, such data does not yet exist for non-IgE mediated milk and egg allergy. In addition, tolerance to baked soya and processed wheat, has not been established in IgE-mediated allergies and to the best of our knowledge we are the first center that reports using this HIP approach for the reintroduction of these allergens in non-IgE mediated soya and wheat allergy. This study, therefore, cannot infer any association with tolerance of allergens, but merely describes a method of home re-introduction of allergens for non-IgE mediated allergic patients. A survey on the use of the milk ladder approach found that many centers in the UK and United States were already using a home-based reintroduction due to limited hospital resources for inpatient admissions and concluded that the development of safe reintroduction protocols may help in many countries with limited in-hospital challenge resources for non-IgE mediated allergies (15).

This study has three main limitations. The first is related to the decision of when to commence the HIP. Whilst the aim was to do this 4–8 weeks after allergen elimination, we did utilize an individual approach which considered ongoing symptoms and general health of the child, reflecting the complexity of the cohort from a tertiary referral center. This however meant, that some children did not have the allergen reintroduced for several months after elimination. One could therefore argue that some children may not have been allergic to the food eliminated in the first instance (as this was not confirmed immediately after diagnosis) or had developed tolerance to the allergen in the period prior to the home reintroduction. Future studies should therefore aim to do home reintroduction at set/specific time intervals after diagnosis. Although symptoms were monitored by an experienced gastroenterologist and dietitian, we were still reliant on subjective feedback regarding symptoms. Where results were ambiguous, we did repeat the HIP and would recommend other centers using home introductions to repeat home reintroductions whenever the outcome is not clear.

The next main limitation of the study is related to the nature of the reintroductions being performed at home: potential bias from reliance on parental reporting and monitoring of symptoms and the lack of clinically validated tools to assess failure/pass of the HIP. The gold standard for diagnosis of delayed non-IgE mediated allergies remains a supervised double blind food challenge, which rules out bias relating to parental interpretation of symptoms and the expectations from researchers. Although we acknowledge that this remains the ideal, we also need to be investigating methods that offer healthcare professionals a more practical approach in clinical practice, as performing double blind challenges are difficult in delayed allergies, in particular in resource poor areas. Although home introduction is practical, performing these over a longer period of time can be influenced by confounding factors, increasing the need for repeating introductions if results are ambiguous. Future studies should aim to validate tools to aid healthcare professionals in better assessing passed/failed home re-introduction, investigate home introduction approaches on tolerance and assess quality of life: comparing the graded approach (beginning with baked milk) to rapid introduction using unprocessed/unbaked foods and doing this at a set time interval.

The third limitation is the fact that this study took part at a tertiary specialist center, which may represent the more complex spectrum of non-IgE mediated allergies. The presented data may therefore not reflect reintroduction of allergens of patients with milder non-IgE mediated allergies, where this may occur earlier and may require less attempts. Future studies are required to establish this in patients in primary and secondary care settings.

## Conclusion

This is the first study investigating HIPs for children with non-IgE mediated gastrointestinal allergies using the food ladder approach, where the final dose was based on a combination of the National Diet and Nutrition Survey results and existing guidelines for FPIES. No serious adverse events were reported in our cohort and we have shown that a significant number of children were fully tolerant to wheat, soya, egg and milk between 9 and 18 months of age following re-introduction. The HIPs itself will require further validation from other centers and most importantly the success of HIP should be assessed with a more timely reintroduction of allergens.

## Data Availability Statement

The datasets presented in this article are not readily available because Authors have moved away from the primary producing centre, which has retained the dataset. Requests to access the datasets should be directed to r.meyer@imperial.ac.uk.

## Ethics Statement

The studies involving human participants were reviewed and approved by NRES Bloomsbury London - 11/LO/1177. Written informed consent to participate in this study was provided by the participants' legal guardian/next of kin.

## Author Contributions

RM: study design, supervision of study, development of challenge protocols, write up of article, and RM data analysis. CD: data collection, development of challenge protocols, and write up of the article. RD performed data analysis and write up of article. A-KS: development of challenge article. HG, AC-L, and ME: data collection and write up of article. R-XF: write up of article. NS: study group lead, study design, and write up of article. All authors contributed to the article and approved the submitted version.

## Funding

Funding was obtained from Great Ormond Street Hospital Charity.

## Conflict of Interest

RM does academic lectures for Nestle, Mead Johnson, Abbott and Danone/Nutricia NS has since performing and completing the research moved to Reckitt Benckiser. The remaining authors declare that the research was conducted in the absence of any commercial or financial relationships that could be construed as a potential conflict of interest.

## Publisher's Note

All claims expressed in this article are solely those of the authors and do not necessarily represent those of their affiliated organizations, or those of the publisher, the editors and the reviewers. Any product that may be evaluated in this article, or claim that may be made by its manufacturer, is not guaranteed or endorsed by the publisher.
