# Supplementary material for: The Challenge of Home Allergen Re-introductions Using the Ladder Approach in Children With Non-IgE Mediated Gastrointestinal Food Allergy
Source: Front Allergy. 2021 Sep 10;2:721686. doi: 10.3389/falgy.2021.721686 (PMC8974734; doi:10.3389/falgy.2021.721686)
Supplement: Supplementary file 1 [file Table_1.DOCX]

Appendix 1: Summary of final amounts of food allergen protein aimed for in the home introduction

| **Allergen** | **Children 8 month-5 years** | **Children 5 - 12 years** | **Children 12-16 years** |
| --- | --- | --- | --- |
| **Cow’s Milk** | 10-15g cow’s milk protein daily  **2-3 portions**  150 ml full cream milk OR  200 ml standard infant formula milk  100g full cream yoghurt  20 g cheese | 15-20g cow’s milk protein daily  **3 portions**  150ml semi skimmed milk  100g low fat yoghurt  30g cheese | 20 - 25g cow’s milk protein daily  **3 portions**  200ml semi skimmed milk  150g low fat yoghurt  30g cheese |
| **Soya** | 8-10g soya protein daily  **2-3 portions**  150 ml soya milk or  200 ml soya infant formula  80-100g soya yoghurt  80-100g custard dessert  20g soya cheese  40 g tofu | 10-15g soya protein daily  **3 portions**  150 ml soya milk  100g soya custard dessert  100g soya yoghurt  30 g soya cheese  80 g tofu | 15-20g soya protein daily  **3 portions**  200 ml soya milk  150g custard dessert  150g soya yoghurt  30g soya cheese  100 g tofu |
| **Wheat** | 5-8 g wheat protein daily  **2-3 portions**  1 Weetabix§  1-2 slices bread  ½ cup cooked wheat pasta | 8-12g wheat protein daily  **2-3 portions**  2 Weetabix  2 slices of bread  1 cup cooked wheat pasta | 12-18g wheat protein daily  **3 portions**  2 Weetabix  2-3 slices bread  2 cups cooked wheat pasta |
| **Egg** | 5-10g egg protein daily  **1 portion**  1 medium egg  Unlimited manufactured goods containing egg protein* | 8- 12g egg protein daily  **1-2 portions**  1 large egg  Unlimited manufactured goods containing egg protein | 8- 15g egg protein daily  **1-2 portions**  1 large egg  Unlimited manufactured goods containing egg protein |

§wheat based UK breakfast cereal

*biscuits, egg pasta, cakes and mayonnaise

Appendix 2: Home introduction of milk using the cumulative ladder approach

| **Days** | **Stages**  **(Introductions are cumulative∝)** | **Total Daily Milk protein** | | |
| --- | --- | --- | --- | --- |
|  |  | **8 months -5 years** | **5-12 years** | **12 – 16 years** |
| **Day 1-3** | **Baked milk in biscuits/muffin φ** | < 0.5 g | < 1 g | 1-2 g |
| **Day 4-6** | **Increased portion of baked milk in muffin/biscuits** | 1 g | 2 g | 2-3 g |
| **Day 7-9** | **Hard highly fermented Cheese*** | 6-8 g | 8-10 g | 10-12 g |
| **Day 10-12** | **Yoghurt or soft cheese§** | 8-12 g | 12-15 g | 15-20g |
| **Day 13-15** | **Infant Formula or fresh cow’s milk♯** | 10 -15g | 15-20g | 20-25g |

∝Once a stage is tolerated the foods need to be continued as part of the child’s diet – protein content therefor increases cumulatively with each step

φCommercial names of biscuits with that amount of milk protein given or muffin recipe

*Parmigiano Reggiano cheese

§Spreadable soft cheese

♯ Semi-skimmed > 1 year of age for challenge period and then switched to full cream milk depending on the age of the child

Average protein content of milk products used:

Full cream milk 3.4 g/100 ml protein

Semi skimmed milk 3.6 g/100 ml protein

Cheese 34 g/100 g protein

Natural full cream yoghurt 3.6 g/100 ml protein

Appendix 3: Home introduction of soya using the ladder approach

| **Days** | **Stages**  **(Introductions are cumulative∝)** | **Total Daily Soya protein** | | |
| --- | --- | --- | --- | --- |
|  |  | **1 - 5 years** | **5-12 years** | **12 - 16 years** |
| **Day 1 -3** | **Soya as an ingredient in small amounts**  **Bread with soyaΦ** | < 0.5 g | 0.5-1g | 1g |
| **Day 4-6** | **Soya as an ingredient in small amounts, increasing portions**  **Bread with soya** | 0.5- 1g | 1-1.5g | 1.5- 2g |
| **Day 7-9** | **Soya products**  **Soya cheese**  **Tofu** | 3-5g | 4-6g | 5-8g |
| **Day 10-12** | **Soya products**  **Soya custard dessert, Soya yoghurt** | 5g | 10g | 10-15g |
| **Day 13-15** | **Soya milk**  **Fresh Soya milk or Soya formula** | 8-10g | 10-15g | 15g -20g |

∝Once a stage is tolerated the foods need to be continued as part of the child’s diet – protein content therefor increases cumulatively with each step

ΦCommercial names of bread with that amount of soya was given to parents or muffin recipe

Average protein content of soya products:

Soya milk 3g/100 ml

Soy milk for toddlers 2.5g/100 ml

Natural soya yoghurt 4g/100 ml

Soya custard 3g/100 ml

Soya cheese 10.4g/100 g

Tofu 8g/100g

Appendix 4: Home introduction of wheat using the ladder approach

| **Days** | **Stages**  **(Introductions are cumulative∝)** | **Total Daily Wheat protein** | | |
| --- | --- | --- | --- | --- |
|  |  | **1 - 5 years** | **5-12 years** | **12 - 16 years** |
| **Day 1 - 2** | **Wheat in small amountsψ**  **Whole wheat bread or**  **pasta or**  **Weetabix℘** | < 0.5 g | 0.5- 1g | 1-2 g |
| **Day 3 - 4** | **Wheat in increasing portions**  **Bread/pasta/Weetabix** | 3g | 3-5g | 5g - 8g |
| **Day 5 - 7** | **Wheat as main carbohydrate source**  **Pasta/bread/Weetabix** | 4g -6g | 6g -8g | 8g -12g |
| **Day 8 - 10** | **Increasing portion of wheat as main source of carbohydrate**  **Pasta/bread/Weetabix** | 6g -10g | 8g – 12g | 12g – 18g |

∝Once a stage is tolerated the foods need to be continued as part of the child’s diet – protein content therefor increases cumulatively with each step

℘ Wheat based breakfast cereal

ψ Muffin recipe was not used for the wheat home reintroduction

Average protein content of wheat products:

Weetabix 2.25g/square

Whole wheat bread 3.9g/30g slice

Cooked pasta 6g/100 g

Appendix 5: Home introduction of egg using the cumulative ladder approach

| **Days** | **Stages**  **(Introductions are cumulative∝)** | **Total Daily Egg protein** | | |
| --- | --- | --- | --- | --- |
|  |  | **8 months- 5 years** | **5-12 years** | **12-16 years** |
| **Day 1-3** | **Baked egg in small amounts**  **Muffin or commercial cakes φ** | 0.5-1 g | 1 g | 1-2g |
| **Day 4-6** | **Baked egg in increasing portions** | 1g - 2g | 2g - 4g | 3g -5g |
| **Day 7-9** | **Well cooked eggδ**  **Small egg – 50-80 g**  **Medium egg – 80-100 g**  **Large egg – ≥ 100 g** | 5g -10g | 8 - 12g | 8-15g |
| **Day 10-12** | **Scrambled cooked egg**  **Small egg – 50-80 g**  **Medium egg – 80-100 g**  **Large egg – ≥ 100 g** | 5g -10g | 8g –12g | 8-15g |

φ Recipe provided or commercial names of cakes given to parents to match protein content of egg

∝Once a stage is tolerated the foods need to be continued as part of the child’s diet – protein content therefor increases cumulatively with each step

δ Boiled egg for 10 minutes or scrambled and then placed in microwave to ensure no loosely cooked areas

Average protein content of egg:

Medium-large egg 6 g

Appendix 6: Muffin recipe for home reintroduction

Ingredients:

**225g (8oz) plain flour** or **gluten free flour and ½ teaspoon of Xanthan gum**

Pinch of salt

1 tbsp baking powder

55g (2oz) caster sugar (for younger children, increase fruit to 130 g and reduce sugar to 1 oz)

55g (2oz) dairy free spread

**150ml full cream cow’s milk (if the muffin recipe was done for egg introduction, the cow’s milk was replaced with a suitable plant-based milk**

2 tsp lemon juice

½ tsp vanilla extract

**1 egg beaten** **or 1 tbsp potato flour mixed with 3 tbsp water to replace 1 egg**

100g (3.5oz) fruit (any mashed- not pureed)

1. Line 6 large or 10 small muffin tin with paper cases or grease the tins well. Preheat oven to 200C (Gas 6)
2. Melt the butter or margarine in a saucepan. Remove from heat and leave to cool.
3. Sift the flour, salt, baking powder, into a bowl. Stir in the sugar.
4. Make a well in the centre; pour in the milk, lemon juice, vanilla extract and egg. Fold in the mixture quickly to combine, but don’t worry if there are a few lumps. Fold in the blueberries carefully into the mixture.
5. Spoon the mixture into the prepared tins or cases.

**Milk protein:** 0.85 g in 6 large muffins and 0.51 g 10 small muffins

**Egg protein:** 1 g in 6 large muffins and 0.6 g in 10 small muffins

**Soya protein:** 0.75 g in 6 large muffins and 0.45 in 10 small muffins

**Wheat protein:** 3.87 g in 6 large muffins and 2.3 g in 10 small muffins

Appendix 7

Follow-up Questionnaire when home introduction protocol has started

| **Symptoms:** please indicate which symptoms your child experiences? | | |
| --- | --- | --- |
|  | **Please indicate the severity of the symptoms:**  none mild moderate severe |  |
| **Diarrhoea:** (loose, watery, frequent stools) | 0 1 2 3 4 5 |  |
| **Constipation:** (excessive straining, low frequency, hard stools) | 0 1 2 3 4 5 |  |
| **Vomiting:** (unexplained vomiting, often associated with abdominal pain) | 0 1 2 3 4 5 |  |
| **Rectal bleeding:** (bleeding from the bottom when passing a stool, fresh blood) | 0 1 2 3 4 5 |  |
| **Abdominal pain:** (affects daily functioning such as school and sleep) | 0 1 2 3 4 5 |  |
| **Wind/flatus:** (excessive burping and painful wind) | 0 1 2 3 4 5 |  |
| **Bloating:** (bloated/extended stomach that is hard when pressed) | 0 1 2 3 4 5 |  |
| **Screaming/back arching:** (screaming associated with back arching and kicking legs out straight) | 0 1 2 3 4 5 |  |
| **Food aversion:** (pushes away food, gags, holds food in mouth, spits/throws food, cries during meals) | 0 1 2 3 4 5 |  |
| **Any other symptoms:** |  |  |
